# Harnessing the power of artificial intelligence and robotics impact on attaining competitive advantage for sustainable development in hospitals with conclusions for future research approaches

**DOI:** 10.3205/dgkh000470

**Published:** 2024-03-15

**Authors:** Narasingappa Pavithra, Noor Afza

**Affiliations:** 1 Department of Studies in Research and Business Administration, Tumkur University, Tumkur, Karnataka, India

**Keywords:** artificial intelligence, robotics, competitive advantage, hospitals, sustainable development

## Abstract

Artificial intelligence (AI) and robotics have emerged as game-changing technologies with the potential to revolutionize the healthcare industry. In the context of hospitals, their integration holds the promise of not only improving patient care but also driving competitive advantage and fostering sustainable development. This review paper aims to explore and evaluate the impact of AI and robotics applications on attaining competitive advantage and promoting sustainable development in hospitals, examines the current landscape of AI and robotics adoption in healthcare settings and delve into their specific applications within hospitals, including AI-assisted diagnosis, robotic surgery, patient monitoring, and data analytics. A key finding is the insufficient use of KI to date in terms of promoting sustainable development in hospitals. Furthermore, attempts to analyze the potential benefits and challenges associated with these technologies in terms of enhancing patient outcomes, operational efficiency, cost savings, and differentiation from competitors. Drawing upon a comprehensive review of the existing literature and case studies, this paper provides valuable insights into the transformative potential of AI and robotics in hospitals.

## Abstract (de)

Künstliche Intelligenz (KI) und Robotik haben sich als bahnbrechende Technologien erwiesen, die das Potenzial haben, die Gesundheitsbranche zu revolutionieren. Im Kontext von Krankenhäusern verspricht ihre Integration nicht nur eine Verbesserung der Patientenversorgung, sondern auch einen Wettbewerbsvorteil und eine nachhaltige Entwicklung. Diese Übersichtsarbeit zielt darauf ab, die Auswirkungen von KI- und Robotikanwendungen auf die Erzielung von Wettbewerbsvorteilen und die Förderung einer nachhaltigen Entwicklung in Krankenhäusern zu untersuchen und zu bewerten. Das Review untersucht die KI- und Robotikanwendungen im Gesundheitswesen und befasst sich mit deren spezifischen Anwendungen in Krankenhäusern einschließlich KI-gestützter Diagnose, Roboterchirurgie, Patientenüberwachung und Datenanalyse. Ein wichtiges Ergebnis ist die bisher ungenügende Nutzung von KI in Hinblick auf die Förderung der nachhaltigen Entwicklung in Krankenhäusern. Darüber hinaus wird versucht, die potenziellen Vorteile und Herausforderungen zu analysieren, die mit diesen Technologien in Bezug auf die Verbesserung der Patientenergebnisse, der betrieblichen Effizienz, der Kosteneinsparungen und der Differenzierung von der Konkurrenz verbunden sind. Auf der Grundlage einer umfassenden Überprüfung der vorhandenen Literatur und von Fallstudien bietet das Review weiterführende Einblicke in das transformative Potenzial von KI und Robotik in Krankenhäusern.

## Introduction

The integration of artificial intelligence (AI) and robotics in the healthcare industry has revolutionized the way hospitals deliver care. These technologies have shown tremendous potential in enhancing patient outcomes, improving operational efficiency, reducing costs, and driving competitive advantage [1]. Hospitals have been under increasing pressure to innovate and differentiate themselves from competitors while also addressing the challenges of sustainability and social responsibility [2]. The use of AI and robotics presents an opportunity to achieve these goals simultaneously, by harnessing the power of technology to deliver sustainable, equitable, and high-quality healthcare [3].

This review aims to explore and evaluate the impact of AI and robotics on attaining competitive advantage and promoting sustainable development in hospitals. Specifically, we examine the current landscape of AI and robotics adoption in healthcare settings, the specific applications of these technologies in hospitals, and the potential benefits and challenges associated with their use [4]. Moreover, we analyze their contribution to sustainable development goals, including environmental sustainability, social responsibility, ethical use of technologies, and equitable access to healthcare.

## Method

Through an extensive literature review is carried to get insights of Artificial intelligence and Robotics. The paper is descriptive in nature and provides valuable insights into the transformative potential of AI and robotics in hospitals. The findings and recommendations can guide hospital administrators, policymakers, and healthcare professionals in leveraging these technologies to attain competitive advantage and foster sustainable development, ultimately enhancing patient care and outcomes in the rapidly evolving healthcare landscape.

## Results

Despite the growing body of literature on the impact of artificial intelligence (AI) and robotics in hospitals, there are still several research gaps that need to be addressed. This review has identified the following research gaps:

### Limited focus on long-term sustainability

While the benefits of AI and robotics in hospitals for competitive advantage and sustainable development are well-documented, there is a lack of studies that delve into the long-term sustainability implications [5]. Future research should explore the durability and adaptability of these technologies, considering their lifecycle, maintenance, and scalability to ensure continued competitive advantage and sustainable outcomes.

### Ethical considerations

Although ethical considerations are briefly discussed, further research is needed to delve deeper into the ethical implications of AI and robotics in hospitals. This includes addressing issues related to bias in algorithms, privacy and security concerns, the responsibility of decision-making algorithms, and the impact on patient-doctor relationships [6]. Exploring these ethical dimensions will contribute to the responsible implementation and adoption of AI and robotics in hospitals.

### Human-machine collaboration

While AI and robotics have shown tremendous potential in augmenting human capabilities, there is limited research on the optimal collaboration between healthcare professionals and these technologies [1]. Understanding the dynamics of human-machine collaboration, identifying the most effective ways to integrate AI and robotics into existing healthcare workflows, and addressing the acceptance and trust issues among healthcare professionals are important areas for future investigation [4] .

### Economic considerations

The economic aspects of adopting AI and robotics in hospitals for competitive advantage and sustainable development require further exploration [7]. Research should focus on cost-effectiveness analyses, return on investment, and financial sustainability models to provide insights into the economic viability and implications of these technologies for hospitals of different sizes and resource settings [8].

### Patient-centered outcomes

While the review highlights the potential benefits for patient outcomes, further research is needed to examine the specific impact of AI and robotics on patient-centered outcomes such as patient satisfaction, patient engagement, and patient-reported outcomes [9]. Understanding how these technologies enhance patient experiences and outcomes will provide valuable insights for healthcare providers and policymakers [10].

Addressing these research gaps will contribute to a more comprehensive understanding of the impact of AI and robotics on attaining competitive advantage and promoting sustainable development in hospitals, ultimately leading to improved healthcare delivery and patient outcomes.

## Discussion

The integration of artificial intelligence (AI) and robotics in hospitals has the potential to significantly impact competitive advantage and sustainable development. This review has explored the various dimensions of this impact, considering the current adoption landscape, specific applications, benefits, challenges, and contributions to sustainable development goals.

The findings of this review highlight the diverse applications of AI and robotics in hospitals. These technologies have been utilized for AI-assisted diagnosis, robotic surgery, patient monitoring, and data analytics, among others [1]. The benefits associated with their adoption are substantial. Improved patient outcomes, enhanced operational efficiency, cost savings, and differentiation from competitors have been reported as key advantages [11]. AI and robotics enable more accurate diagnoses, precise surgical procedures, real-time patient monitoring, and efficient utilization of healthcare resources [5]. These benefits contribute to the competitive advantage of hospitals by attracting patients, improving overall quality of care, and increasing operational effectiveness.

Furthermore, AI and robotics play a crucial role in promoting sustainable development in hospitals. They address environmental sustainability through energy-efficient systems, waste reduction, and smart resource utilization. Additionally, they facilitate social responsibility by promoting equitable access to healthcare and enabling personalized care delivery [12]. Ethical considerations, such as ensuring the responsible use of these technologies and addressing privacy and security concerns, are essential for their sustainable implementation [13].

However, it is important to acknowledge the challenges and risks associated with AI and robotics adoption in hospitals. These include concerns related to data privacy and security, ethical implications, workforce dynamics, and the potential for bias in decision-making algorithms. Addressing these challenges requires collaboration between healthcare stakeholders, policymakers, and technology developers to establish ethical guidelines, ensure transparency, and foster ongoing education and training for healthcare professionals [14].

Based on the findings, several avenues for future research and development can be identified.

### Advanced AI applications

Future research can focus on exploring advanced AI applications in hospitals to further enhance patient care and operational efficiency. This includes the integration of natural language processing and deep learning algorithms for more accurate diagnosis, predictive analytics for personalized treatment plans, and AI-powered virtual assistants to improve patient interactions and support healthcare professionals [9].

### Human-robot interaction

As robotics becomes more prevalent in healthcare settings, there is a need for research on human-robot interaction. Future studies can explore the design and development of socially intelligent robots that can effectively collaborate with healthcare professionals and provide personalized care to patients [15]. Understanding the dynamics of trust, acceptance, and communication between humans and robots is crucial for successful implementation.

## Ethical frameworks and guidelines

Given the ethical implications associated with AI and robotics in healthcare, the development of comprehensive ethical frameworks and guidelines is imperative. Future research can focus on establishing standardized ethical guidelines for the responsible use of AI and robotics in hospitals. This includes addressing issues related to privacy, data security, algorithmic bias, and the responsible deployment of autonomous systems [14].

### Long-term impact assessment

To ensure the long-term sustainability of AI and robotics in hospitals, future research should focus on assessing the long-term impact of these technologies. This includes evaluating their effects on patient outcomes, healthcare provider satisfaction, cost-effectiveness, and environmental sustainability over extended periods. Longitudinal studies and economic evaluations can provide valuable insights into the sustainable benefits and challenges of AI and robotics adoption [4].

### Collaborative healthcare ecosystems

Research can explore the development of collaborative healthcare ecosystems, where hospitals, technology developers, policymakers, and patients work together to maximize the potential of AI and robotics. This involves fostering interdisciplinary collaboration, data sharing, and standardization of technologies to promote interoperability and seamless integration across healthcare systems [3].

### Addressing healthcare disparities

Future research should address the potential of AI and robotics to bridge healthcare disparities. By focusing on equitable access, affordability, and cultural considerations, researchers can investigate how these technologies can help overcome existing disparities in healthcare delivery and improve health outcomes for underserved populations [1].

By addressing these future research directions, healthcare organizations, policymakers, and technology developers can continue to harness the power of AI and robotics in hospitals, driving innovation, enhancing patient care, and promoting sustainable development.

## Conclusion

This review highlights the transformative potential of AI and robotics in hospitals for attaining competitive advantage and promoting sustainable development. By leveraging these technologies, hospitals can improve patient care, enhance operational efficiency, reduce costs, differentiate themselves from competitors, and contribute to sustainable development goals. However, careful consideration of ethical, social, and operational aspects is necessary for successful implementation. By embracing AI and robotics responsibly, hospitals can embrace the future of healthcare, driving innovation, and improving patient outcomes.

## Notes

### Competing interests

The authors declare that they have no competing interests.
